# Inflated Applicants: Attribution Errors in Performance Evaluation by Professionals

**DOI:** 10.1371/journal.pone.0069258

**Published:** 2013-07-24

**Authors:** Samuel A. Swift, Don A. Moore, Zachariah S. Sharek, Francesca Gino

**Affiliations:** 1 Haas School of Business, University of California, Berkeley, California, United States of America; 2 CivicScience, Pittsburgh, Pennsylvania, United States of America; 3 Harvard Business School, Harvard University, Cambridge, Massachusetts, United States of America; Cardiff University, United Kingdom

## Abstract

When explaining others' behaviors, achievements, and failures, it is common for people to attribute too much influence to disposition and too little influence to structural and situational factors. We examine whether this tendency leads even experienced professionals to make systematic mistakes in their selection decisions, favoring alumni from academic institutions with high grade distributions and employees from forgiving business environments. We find that candidates benefiting from favorable situations are more likely to be admitted and promoted than their equivalently skilled peers. The results suggest that decision-makers take high nominal performance as evidence of high ability and do not discount it by the ease with which it was achieved. These results clarify our understanding of the correspondence bias using evidence from both archival studies and experiments with experienced professionals. We discuss implications for both admissions and personnel selection practices.

## Introduction

The National Association of Colleges and Employers reports that 66 percent of employers screen candidates by grade point average (GPA), and 58 percent of employers indicated that a GPA below 3.0 all but eliminates a candidate's chances of being hired [1]. Academic grades, after all, test the mental ability central to predicting job performance [2]. However, by setting a cutoff GPA for all candidates, employers implicitly assume that a grade from one school is equivalent to a grade from another. This is a problematic assumption because universities vary considerably in their grading standards; otherwise similar universities display large differences in the grades they award their students [3]–[7]. Accounting for variation in grade distributions between institutions represents a special case of a more general problem: how to make attributions about performance while considering both the performer's abilities and the difficulty of the task.

Organizations encounter this vexing attribution problem routinely. For example, who should be promoted: the marketing manager who has presided over excellent sales growth for a cutting-edge product in a hot market segment or the marketing manager who has produced modest sales growth for a product near the end of its life cycle in a shrinking market segment? To what degree is each product's sales a result of the manager's abilities and to what degree is it a consequence of the situation? Indeed, corporations regularly promote workers with strong records of achievement into jobs that require new skills only to see them fail [8]–[10].

Although decisions like these are common in organizations, anecdotal evidence suggests that managers are not able to account sufficiently for situational influences on performance when they make predictions regarding the likelihood of success of their employees in a new job [11]. This anecdotal evidence is supported by psychological research on attribution which suggests that people have trouble making this type of attribution accurately [12]. Attributional judgments are predictably impaired by the correspondence bias [13], a tendency so widespread that Ross [14] declared it the fundamental attribution error. One way in which organizations make this error is by assuming that performance in one domain will predict performance in another domain. Hogarth and Kolev, for instance, present evidence suggesting that CEOs who are good at playing golf are paid more. In fact, however, they actually perform worse in their jobs, perhaps because they are spending too much time on the golf course [15].

Another way in which organizations fall victim to this bias is when they rely on dispositional attributions for what are actually situational effects. It leads managers to explain workers' performance in terms of their inherent abilities despite the undeniable influence of their social surroundings and institutional constraints [16], [17]. This natural tendency may have contributed to interest in the search for the individual dispositions that make the best leaders [18]–[20], a research approach that is now regarded as misguided [21]. There is, of course, a parallel literature in the field of entrepreneurship that sought to identify the traits that predispose individuals to entrepreneurial success [22], [23]. This too is generally regarded as having been a failure [24], [25]. Both research agendas sought to identify the distinctive personality characteristics that contribute to success and both have had disappointing results. The alternative approach assumes that success is determined in powerful ways by social settings and the opportunities they afford or the constraints they impose on leaders, entrepreneurs, and the rest of us [26]–[28].

### The correspondence bias

How should organizations assess employee dispositional ability given noisy performance data that is confounded with situational difficulty? The answer comes from Kurt Lewin's [29] attributional equation: *Behavior*  =  *f*(*Disposition*, *Situation*). In other words, an individual's performance is a joint function of both the individual's disposition (traits, abilities, and predilections) and the situation in which the individual finds him- or herself. Gilbert [12] proposed the following simplified specification of Lewin's attributional equation: *Behavior*  =  *Disposition* + *Situation*. Alternatively, if our goal is to understand the individual's disposition: *Disposition*  =  *Behavior* – *Situation*. Gilbert's simplification holds when performance is the sum of an individual's ability and task ease. Using this equation, we can infer an individual's disposition so long as we can measure both their performance and how that performance was affected by the situation. Let us say, for example, that an Olympic figure skating judge is exactly two tenths of a point more lenient than the other judges are. Any time the lenient judge is on the panel, those skaters' performance received a better evaluation. Computing the scores that skaters deserved to receive is easy: simply take two tenths of a point off from the lenient judge's rating.

The correspondence bias interferes with the simple logic of the attributional equation by ascribing too little influence to the situation and too much to the individual's disposition [21], [30]. For instance, this bias would result in skaters being judged as better after having been evaluated by lenient judges. There is evidence that managers do indeed tend to favor dispositional attributions for employee performance [17], [31]. However, these studies cannot specify whether such attribution represents accuracy or bias because the research designs did not afford accurate assessments of the actual causes of performance. The laboratory evidence on the correspondence bias, for its part, is constrained by a different issue. Because no participant has objective information on both performance and situational influences on performance, it may be unrealistic to expect that anyone will be able to make the optimal judgment. Indeed, whenever people have better information about performance than about the situation, it should be no surprise that they neglect to consider the situation.

Consider, for example, the classic study by Ross, Amabile, and Steinmetz [32]. In it, participants were randomly assigned to the roles of quiz-master, quiz-taker, or observer. Quiz-masters came up with quiz questions to which they knew the answers. The quiz-takers, on the other hand, did not know the answers to all of the questions. Observers, who only observed one quiz-master and one quiz-taker, faced the difficult task of determining which of the two was more knowledgeable. On average, observers rated the randomly assigned quiz-masters as being more knowledgeable, suggesting that they based their judgments on behavior without accounting for the impact of the role. But to suggest that observers should have ignored their observations and simply rated quiz-master and quiz-taker equally knowledgeable is unrealistic because it neglects the information present in the quiz game. If the questions were all easy and the quiz-taker knew the answers to none of them, then it would not be crazy to infer that this particular quiz-master was indeed more knowledgeable than this particular quiz-taker. The fact that the quiz-master and quiz-taker must be equally knowledgeable, on average (thanks to their random assignment to roles), is of little help determining whether a particular quiz-master is more or less knowledgeable than a particular quiz-taker.

To assess the strength of the situational advantage conferred on quiz-masters, individual participants would have to know what percentage of questions the average quiz-taker got right in this situation. However, because participants in correspondence bias experiments do not receive the information that would allow them to assess the strength of the situation, perhaps it should come as no surprise that the situation is neglected in their attributional calculus [33], [34]. In this paper we offer a new approach in which participants have excellent information about situational influences. They are asked to make inferences about a target's disposition using information about situation and behavior that is directly comparable and in equivalent terms. This approach allows us to advance our understanding of the correspondence bias by eliminating the concerns of demand and prior beliefs while also enabling us to calculate the quantitative degree to which they neglect this information.

## Experiments

In this paper we test for the correspondence bias in selection decisions and ask how easy it might be to correct it. Previous research has shown that college students facing simulated admissions tasks in the lab favored applicants from favorable situations [35]. We present four studies that extend the implications of prior evidence by showing that attribution errors in selection decisions affect decisions made by experts, in field settings, and across domains. In each, we examine situations in which the difficulty of a prior environment influences candidates' performance and consequently affects how they are evaluated by decision-makers. For each study we report how we determined our sample size, all data exclusions (if any), all conditions, and all measures in the study. First, we examine the decisions made by admissions professionals asked to differentiate between academic excellence and varying grading norms in a controlled experimental study. Next, we extend the lab paradigm to the business context and test the decisions made by executives evaluating the performance of employees in varying business climates. Study 3 addresses alternative explanations which may arise from the applied contexts in the other studies by testing the hypotheses in experimental game. Finally, we return to the admissions context to present the results of an analysis of archival data documenting the decisions made by graduate business schools.

In each of these studies, decision-makers are faced with the challenge of evaluating candidates' aptitude (or disposition) by observing performance and attempting to account for the varying situations. We hypothesize that, consistent with the correspondence bias, candidates from favorable situations will be evaluated more positively and selected more frequently than their counterparts with equally commendable dispositions.

To illustrate the analysis that tests this hypothesis, let us consider an example. Imagine that you are considering promoting one of two executives. Executive A oversees Division A, with sales of $100 million. Executive B, on the other hand, oversees Division B, which has sales of $110 million. Executive A brought Division A up from $90 million while Executive B presided over Division B's increase from $108 to $110 million over the same period of time. It probably makes more sense to consider each executive's performance as a change from previous performance. Every dollar in sales that the divisions were already making before the executives showed up should reduce the amount of credit they receive for sales accrued during their tenure. We expect, then, that nominal performance will matter. It should, however, be discounted by the ease with which it was achieved, and this discounting should be roughly equal in size but opposite in sign. An assessment of the two executives should heavily weight the change in sales each has achieved, and should almost completely discount the total sales each wound up delivering.

### Study 1 – Admissions Professionals in the Lab

We put experienced admissions professionals into a fictional version of their professional roles of evaluating applicants and deciding whether to admit them. To test whether the candidate's success in the admissions process was predicted by the grading norms of their alma maters, we gave participants a simplified admissions decision task with two main pieces of information on each of nine candidates: their GPAs and the distribution from which each GPA came. Each factor was manipulated in three levels, creating a 3 (GPA above average, average, or below average) ×3 (Average GPA at alma mater: high, medium, or low) full factorial design within subjects. Average GPAs were operationalized as 3.6 (high), 3.0 (medium), or 2.4 (low) to generally correspond to the range of grading norms observed in U.S. undergraduate institutions. Performance relative to these means was operationalized as candidate GPAs that were equal to the average, 0.3 points above it, or 0.3 points below it. Because this design creates artificially round numbers, each figure in the stimuli was randomly adjusted to within ±0.02 of the specified value.

Participants knew that the nine fictional institutions from which the nine applicants came did not differ in quality or selectivity. Grading norms at the different institutions were therefore uninformative with respect to student quality. We predicted that our expert participants would rightly reward candidates with above-average GPAs with more positive evaluations and higher probability of admission. The more interesting hypothesis is that there would also be a positive main effect of average GPA despite the irrelevance of this factor to the underlying aptitude of the applicant. This hypothesized effect would be consistent with a judgment process that neglects to discount GPAs sufficiently in light of the ease with which they are achieved.

#### Method

The Carnegie Mellon University institutional review board reviewed and approved the study design, materials, compensation, and recruitment technique. Participants completed written documentation of informed consent before participating.

Participants were all 23 responding members of the staff of the undergraduate admissions office at a selective university in the United States. Participants were recruited by e-mail to a website through which the entire study was conducted. As compensation, participants were entered into a lottery in which they stood a 1 in 10 chance to win $100. Of the participants, 61 percent were male, the average age was 34 years, and the mean professional experience in college admissions was over 5 years.

Participants read the following instructions:

*“In this exercise, you will be playing the role of a member of the admissions committee at a selective MBA program. You are selecting students who would like to obtain masters degrees in business administration. Your most important goal is to select the best candidates from among the applicants. In general, you usually have space to admit about half the applicants.*

*You will see the applications of nine hypothetical students. The set of applicants that you will review all graduated from colleges of similar quality and selectivity. Please review each applicant carefully in order to assess the quality of their prior academic performance in college. Please review one candidate at a time. Answer the questions about each candidate before turning the page to read about the next candidate.”*

Participants received one page of information about each candidate from which to make their judgments. The information presented for each candidate included the candidate's GPA and the average GPA at their alma mater. To emphasize the candidates' performance relative to their peers, the difference between their GPA and the average was presented in the statement, “Candidate 1's GPA is [above/below] the college average by [difference].”

To add richness to the stimuli, participants also read a short transcript of ten recently completed classes. For each of the classes, the candidate's grade was listed along with the average grade given in that class. Grades were generated for each class such that the mean of the candidate's grades were equal to their GPA and the mean of the average grades was equal to the college's average GPA. The individual class grades for each candidate were generated such that the variance was constant across candidates. The names of the fictional colleges and the names of the courses were randomized to avoid creating any confounds. Candidates were presented to each participant in a randomly determined order, which was then rotated across participants in a Latin-squares design.

Participants made two judgments concerning each candidate: how successful the candidate had been in college on a 7-point scale from 1(*“very unsuccessful”*) to 7 (*“very successful”*), and how likely they would be to admit them (as a probability between 0% and 100%). After reviewing and evaluating each candidate individually, participants were asked to select four of the nine candidates for admission. Participants were permitted to review summary information for each candidate as they made their admissions decisions.

After participants finished the experimental portion of the study, they completed a small number of questions assessing their beliefs about how grades should be interpreted in the admissions process. They also told us their ages, years of experience, and job titles.

#### Results

Although each participant produced three responses for each candidate, the results are virtually identical across measures (see Table 1). Accordingly, the three measures were standardized and averaged to create a single measure of the evaluation of each candidate (alpha reliability  = 82).

**Table 1:** Summary of ANOVA results by dependent variable and factor (Study 1).

	Situation (Avg. GPA)			Performance (GPA)			Avg GPA * GPA		
	F (2, 44)	p	ή_p_^2^	F (2, 44)	p	ή_p_^2^	F (4, 88)	p	ή_p_^2^
Success	61.75	<001	0.74	48.02	<001	0.69	3.42	0.012	0.13
Prob. of Accept	58.97	<001	0.79	52.79	<001	0.76	7.74	<001	0.62
Admission	42.43	<001	0.66	52.79	<001	0.76	3.42	<001	0.23
Mean Z-score	94.24	<001	0.81	85.09	<001	0.80	5.88	<001	0.21

This aggregate measure was then subject to a 3 (GPA) ×3 (average GPA at undergrad institution) within-subjects ANOVA. As expected, the results show a strong main effect of GPA, *F*(2,44) = 85.09, *p*<0.001, *η_p_^2^* =  0.80. Those candidates with GPAs above their local averages were much more likely to be admitted (70%) than those with below-average GPAs (12%).

Counter to normative expectations, but consistent with our hypothesis, the average GPA at the candidate's home institution also drove a significant main effect, and even explained as much of the variance in evaluations, *F*(2, 44) = 94.24, *p*<0.001, *η_p_^2^* = 0.81. This main effect suggests that it is just as advantageous to come from a school with high average grades as it is to be above average. Indeed, the effect on the probability of admission is very similar (see Table 2). Candidates from the schools with lower grading norms were admitted 12% of the time while those from the schools with higher average grades were admitted 72% of the time. Note that these differences exist despite the same distribution of performance among applicants from each “type” of school.

**Table 2:** Probability of being accepted by condition (Study 1).

		GPA (Performance)			
		Avg.–0.3	Avg.	Avg. +0.3	Total
Average GPA (Situation)	Low (2.4)	00	09	26	12
	Med (3.0)	04	57	87	49
	High (3.6)	30	91	96	72
	Total	12	52	70	

The interaction between GPA and Average GPA was also statistically significant, but does not explain as much variance in evaluations as the main effects, *F*(4, 88) = 5.88, *p*<0.001, *η_p_^2^* = 0.21. This interaction describes the fact that a better-than-average GPA had its smallest benefit for those who came from institutions with tough grading and low average grades (see Table 2).

Participants also answered two questions intended to shed light on the use of GPA statistics in real admissions decision making. We asked, “For approximately what percentage of candidates is information about their home institution's average GPA available?” Although our experiment afforded decision makers access to full information about the candidates' individual performance as well as the situational influences in their environment, we suspected such data was less readily available in the real world. Indeed, this sample of professional admissions staffers estimated that average grade information was available in only 61.5% of cases. This suggests that the magnitude of the bias measured here is likely to be conservative relative to a naturalistic process, because we provided them with more complete information than they actually have in their jobs.

In response to the question, “When considering information about a candidate, how important is the average GPA at a candidate's home institution compared to their individual GPA?,” the mean response (3.8) was closest to the midpoint “Equally important as the candidate's GPA” of the scale which ranged from “Only the average GPA is important” to “Only the candidate's GPA is important.” This is consistent with what we believe to be the normative decision rule, because the individual's nominal performance and the ease of the situation ought to receive equal and opposite weighting when making dispositional inferences.

#### Discussion

The results of these hypothetical admissions decisions made by experienced professionals suggest that candidates who happen to graduate from schools with higher grading norms may actually have a better chance of being accepted to college or graduate school. This is true independent of their personal performance in that situation. For example, high-performing applicants from low-GPA schools were given lower ratings than under-performing applicants from high GPA schools. This should be cause for concern among stakeholders at institutions with tougher grading norms. The high standards to which the students at these institutions are held may be mistaken for poor performance.

Despite the presence of unambiguous information on the key terms in Lewin's attributional equation, our expert participants in Study 1 were either unwilling or unable to make admissions judgments solely based on applicants' dispositions. The participants may have inferred variation in school quality from the variation in average grades – knowing that this relationship is true to some extent in the real world. We should point out however, that one of the few instructions for the task explicitly described the applicant's alma maters as being of similar quality. Participants may have inferred variation where they were told there was none, but this is not the most parsimonious explanation of the rather dramatic effects we observe. Instead, we argue that the results demonstrate the difficulty people – even experts – have discounting nominal performances in light of situational influences.

### Study 2 – Executives and Promotion Decisions

The decisions made by admissions professionals were consistent with our hypotheses, but we have no reason to believe that these effects are isolated to university admissions. To demonstrate that the effect is not driven by idiosyncratic properties of the admissions domain, we constructed an analogous decision task in the corporate domain. Study 2 seeks to investigate the effect of varying situational difficulty on the evaluation of employees for promotion. Participants, all of whom were working professionals with years of experience with selection and promotion decisions, were asked to evaluate twelve candidates who were up for promotion to a senior management position. We varied the difficulty of the situation and the individual performance of the candidates to examine how the situation affected the dispositional evaluations made by our participants. We hypothesized that managers would favor candidates benefiting from the positive light of easy business situations just as admissions had done for candidates from environments with high average grades.

#### Method

The Carnegie Mellon University institutional review board reviewed and approved the study design, materials, and appropriateness as a class exercise. The IRB granted exemption from documented informed consent as the study involved only minimal risk and participation took place in the context of normal class exercises. Students were not required to participate.

Participants were 129 executive education students (38% female) at a research university in the western United States participating as part of a class exercise. They had on average, 18.0 (SD = 8.6) years of work experience and been involved with an average of 14.2 (SD = 29.6) promotion decisions.

The experiment employed a 2 (situation: easy vs. difficult) ×3 (individual performance: low, medium and high) within-subjects design with two candidates in each cell, resulting in twelve candidates for our participants to evaluate.

Participants evaluated each of the twelve candidates, rating their performance and their suitability for promotion. Participants read a business scenario in which they are the CEO of an airline deciding whom to promote to a senior management position from a set of twelve candidates. The most important measure of their performance is the percentage of flights that leave on time from each of their airports. Moreover, of the twelve airports managed by the candidates, there were two types of airports: Half were historically quite punctual and half that ran late more often.

Task difficulty was manipulated via the historical on-time percentage of flights at each airport. Hard airports had 70% of flights on time; easy airports had 85% on-time flights. Individual performance was manipulated relative to this situational effect: low performance was 5% below the average, medium performance was at the average and high performance was 5% above the average. We varied these artificially round numbers by a few tenths of a percentage point to increase the realism of the scenario.

To ensure that candidates were being evaluated solely based on their on-time performance and not by airport, we counter-balanced each of the possible match-ups between airport and performance and randomized the order in which participants encountered the candidates.

For each candidate, participants were shown the airport's historical on-time performance across ten years: five years preceding the candidate's tenure and the most recent five years under their management. The five-year average on-time percentage was presented with each block of data. Participants answered two questions about each candidate: (a) Performance: “*Please rate the performance of Candidate Name, the manager in charge of operations at this airport.*” on a 7-point scale anchored at 1 (*Very Bad*) and 7 (*Very Good*), and (b) Promotion Worthiness: “*Is Candidate Name worthy of promotion?*” on a 7-point scale anchored at 1 (*Definitely No*) and 7 (*Definitely Yes*).

#### Results

Table 3 summarizes the descriptive statistics for the dependent measures, *Performance* and *Promotion Worthiness*. We averaged the ratings given to the two candidates in each cell for the two measures and submitted them each to a 2 (difficulty) ×3 (performance) repeated-measures ANOVA.

**Table 3:** Ratings of performance and promotion worthiness based on candidate performance and airport difficulty (Study 2).

Situation (Historic on-time %)	Easy ≈ 85%			Difficult ≈ 70%		
Performance	Low	Medium	High	Low	Medium	High
(Relative on-time %)	(5% below)	(average)	(5% above)	(5% below)	(average)	(5% above)
Performance Rating	3.54	4.79	6.14	3.09	4.25	5.55
	(1.10)	(0.85)	(0.91)	(1.03)	(0.80)	(0.94)
Promotion Worthiness Rating	3.18	4.30	5.84	2.71	3.76	5.19
	(1.23)	(1.07)	(0.96)	(1.16)	(1.10)	(1.08)

Average ratings of the candidates appear in each cell. Standard deviations appear in parentheses.

The results for the performance rating revealed a significant within-subjects effect of both situational difficulty, *F*(1,128) = 80.40, *p*<001, *η_p_* ^2^ = 39 and actual performance, *F*(2, 256) = 301.92, *p*<001, *η_p_* ^2^ = 70 on the candidates' performance ratings. The interaction between our two main effects, difficulty × performance, was not significant, *F*(2, 256) = 0.692, *p*>05, indicating that while our participants gave higher ratings to candidates from easy airports, they were consistent in their ratings within each difficulty category.The results for the promotion worthiness rating followed a similar pattern, with significant within-subjects effects for both difficulty, *F*(1, 128) = 73.71, *p*<001, *η_p_* ^2^ = 37, and performance, *F*(2, 256) = 248.539, *p*<001, *η_p_* ^2^ = 66, and a non-significant interaction of difficulty × performance, *F*(2, 256) = 0.480, *p*>05.

#### Discussion

If our participants had not been affected by the correspondence bias, then candidates working at easier airports should not have been awarded higher ratings than their dispositional peers working at more difficult airports. However, what we find is that those who are fortunate enough to be assigned to easy airports are evaluated significantly more positively. We can make strong conclusions with respect to the normative status of participants' decisions, thanks to the control of the experimental paradigm. The experienced professionals who served as participants in the first two studies made consistent errors in deciding whom to admit or promote. They favored those from easy situations, rewarding the fortunate and punishing the unfortunate.

However, legitimate questions remain regarding the degree to which they actually make these same errors in their work. One might be tempted to predict that the effects we observe in the first two studies would be even more dramatic in professional domains that do not afford such clear information about the size of situational effects. On the other hand, it may be that organizations can provide some correctives for individual biases [36]. Hypothetical decisions in an experiment could be limited in their validity as a model of fully consequential and incentivized decision making. We seek to address these limitations by demonstrating convergent evidence in a sample of analogous decisions made in the field.

### Study 3 – Selecting Contestants

To address lingering concerns about potential explanations for the effect we observe, Study 3 sought to rule out alternative explanations having to do with what performance means in different conditions. For instance lenient-grading universities may actually be better, or attract better students, in some ways that we have been unable to measure or identify. Or it may be the case that increasing the on-time performance from 85% is more impressive than increasing it from 70%. To rule out these and related concerns, this study had participants make selection decisions in a context where nominal performance differed because some candidates were graded leniently and some were graded stringently. Crucially, grading leniency was randomly determined and was entirely transparent. Participants' task was to review the performance of a set of contestants in a weight-guessing game and then to select those contestants who they thought would perform best on a subsequent game of the same type.

#### Method

The University of California at Berkeley institutional review board reviewed and approved the study design, materials, compensation, and recruitment technique. Participants completed written documentation of informed consent before participating.

We opened the survey to 200 participants via Amazon's Mechanical Turk web site and paid each person $.50 each to complete an online survey that required an average of 7.5 minutes to complete. The sample size of 200 was chosen based on estimates of yield and effect sizes. First, participants completed an attention check. Next, to provide participants with a direct experience of task difficulty, these individuals saw five photographs of different people, guessed how much each person weighed and then received feedback in the form of the person's actual weight. Next, they reviewed the performance of six putative contestants who had previously played two rounds of the game with different pictures. The task of participants was to review the contestants' performance in round 1 and select who they thought would perform best in round 2. Participants saw each contestant's actual guess along with the actual weights for each of the ten photos in the first round. Answers close enough to the actual weight were designated correct according to their grading standard and appeared highlighted in green. Answers outside this range, which were nominally incorrect, appeared highlighted in red.

Participants were motivated to choose those who were best at weight-guessing because they earned one lottery ticket (toward a $50 prize) each time one of their three chosen contestants answered a question correctly in the second round of the game. The second round of the game consisted of ten pictures, and answers were counted as correct when the guess was within 10 pounds of the person's actual weight.

The experimental manipulation varied how contestants' performance in round 1 was scored. Participants read, “We randomly selected three of the six contestants and held them to a high standard of accuracy: their answers only counted as right if they got within 4 pounds of the truth. For the other half, their answers counted as right if they got within 30 pounds of the truth.” For half of participants, contestants 3, 5, and 6 were held to a high standard. For the other half of participants, contestants 1, 2, and 4 were held to a high standard.

#### Results

We dropped participants from the analysis who failed an attention check and who failed to select three contestants on whom to bet, resulting in 156 participants. Our experimental manipulation significantly affected participants' choices, chi-square(1) = 68.77, *p*<.001. 82% of our participants selected more leniently-graded contestants than stringently-graded contestants, and 81% of the contestants they chose were from those graded leniently. One alternative was that participants selected the three contestants who performed best. The three contestants whose weight estimates generated the lowest mean absolute deviation from the correct answers received, on average, 3.67 out of 10 right in the second round. By comparison, our participants selected contestants who received 3.56 (*SD* = 21) right, and this difference is significantly worse, *t*(155) = −5.80, *p*<001.

#### Discussion

Because participants received full information about contestants' performance, grading criteria, and the arbitrary determination of grading criteria, few alternative explanations for participants' preferences are plausible. Instead, they appear to have responded superficially to the number of items that were counted as correct. Arguably, with sufficient time and motivation, people could have actually assessed contestants' performance themselves and the biasing effect of our experimental manipulation would be reduced or eliminated. We do not, however, claim that the correspondence bias affects every decision, no matter how large and important. If the correspondence bias represents a heuristic or “default” decision mode, that is more than sufficient to produce powerful and pervasive effects on human judgment.

### Study 4 – Actual Admissions Decisions

Studies 1 through 3 demonstrate the difficulty of properly incorporating information about the difficulty of the situation when evaluating performance, at least in the scenarios we created. Of course, even with a sample of professionals one can ask whether the motivation and consequences of real decisions can be emulated in a laboratory design. Moreover, the effect we observe in carefully controlled experimental settings may shrink to insignificance relative to all the other influences in the complexity of real admissions decisions. We move to an archival sample of actual admissions decisions for a final test our hypotheses in the field context.

#### Method

The Carnegie Mellon University institutional review board reviewed and approved the study design as an archival research project without identifiable participants.

We obtained admissions data from four selective (admission rates between 16% and 39%) MBA programs in the United States. These data include each applicant's undergraduate GPA and alma mater, as well as a number of useful control variables such as age, gender, race, national citizenship, years of work experience, standardized test scores (in this case, the GMAT test), and interview performance. Each school provided multi-year data ranging from three to eleven years of coverage and drawing from years between 1999 and 2009. Annual applicant totals per school varied from 1,236 to 3,742, yielding between 5,700 and 20,100 applicants per school across all available years, and a total dataset of 56,796 applicants. Of this set 24,994 were graduates of foreign undergraduate institutions and are excluded from the analyses due to incompatible grading systems.

We collected additional data describing the undergraduate institutions (i.e. school quality, average GPA) from which the applicants had graduated. We began with two measures of school quality. The first, Jack Gourman's ratings of undergraduate institutions is an academically cited continuous measure of the quality of education provided for nearly every U.S. institution [37]. As an additional measure of quality, we used the US News & World Report annual ranking of the US colleges and universities [38]. Although the US News measure of quality is not academically reviewed and does not offer complete transparency in its methodology, it captures some dimensions of school quality – such as perceived prestige and alumni success – that may be important in our model. Perceptions of institution quality may actually be more direct drivers of admissions decisions than the underlying quality Gourman strives to capture As a final, more objective measure of school quality we used the average GMAT score of entering students.

We attempted to collect data on average grades for all institutions represented in our sample of applicants. The data on average grades we collected came from a variety of sources. The largest single source was a publicly available database of average GPAs assembled for the purpose of documenting trends in grade inflation [6], but this source was not comprehensive. The remaining institutions for which we lacked average grade information were contacted directly by email or telephone, as necessary. Many declined to provide us with information about average grades. In the end, we were able to obtain average GPA data from 198 institutions, corresponding to 20,913 applicants or 77% of the domestic applicants in our sample. Average GPA of the applicants' undergraduate institution was the most restrictive variable in our dataset, with the other predictors ranging from 85%–100% complete.

Applicants' performance was measured by their deviation from the average GPA at their undergraduate institution, henceforth: relative GPA. By modeling performance as the difference between those two raw data sources, we were able to directly test our hypothesis that applicants' situations affect their outcomes above and beyond their unique performances in that situation. Relative GPA is a standard unit of performance that can be used to compare the disposition of students across situations. Using relative GPA as our measure of performance solves two problems with model interpretation. First, including both GPA and average GPA in a model complicates their interpretation since average GPA (together with the student's performance) is a component of one's nominal GPA. Second, using relative GPA also avoids problems with logistic regression assumptions because the distribution of nominal GPAs is truncated at 4.0 while relative GPA is normally distributed. Note that this calculation of relative GPA is simply a transformation of known values, not a comparison between scale measurements. As such, it is not threatened by the reliability concerns inherent in difference scores [39], [40].

Interview ratings were standardized within each application year at each admitting school to account for different systems of evaluation. Average GPA at a candidate's undergraduate institution was mean centered before being included in the model. Racial identifications are presented as comparisons to the baseline category of Caucasian, which was the largest group in the sample.

#### Results

The means, standard deviations, and bivariate correlations between applicant level variables are shown in Table 4, while Table 5 reports the same statistics for school level variables. As shown in Table 4, there is, not surprisingly, a positive relationship between applicants' undergraduate grades and admission decisions: Those who were admitted had higher GPAs (*M* = 3.35) than those who were not (*M* = 3.29), *t* (36384) = 13.12, *p*<001. The positive effect of institutional GPAs may be because better universities have higher average grades. Indeed, average grades tend to be positively related to institution quality with correlations between average GPA and other measures of quality ranging from *r* = 18 for the Gourman ratings to *r* = 62 for the US News ratings. Average absolute GPA may in fact be correlated with student ability. A more complete picture, then, is provided by a full multiple regression.

**Table 4:** Tabe 4. Applicant level descriptive statistics and bivariate correlations (Study 3); all correlations are statistically significant (p<001).

	Mean	S.D.	1	2	3	4	5	6	7
1. Male	0.72	0.45							
2. Age	29.8	4.56	15						
3. US Citizen	0.53	0.50	−07	−07					
4. GMAT	669	65.4	14	−16	−08				
5. Interview rating	0.00	0.96	−05	05	05	05			
6. Years of work history	5.35	3.16	08	39	−09	05	07		
7. Undergraduate GPA	3.30	0.39	−15	−21	03	16	04	−13	
8. Admitted	0.31	0.46	−04	03	16	17	38	−02	14

**Table 5:** Undergraduate institution level descriptive statistics and bivariate correlations (Study 3); all correlations are statistically significant (p<.05) unless marked (*).

	Mean	S.D.	1	2	3	4	5	6
1. Gourman quality score	3.5	0.6						
2. US News quality score	61.7	16.1	31					
3. Average entrance SAT	1153	144	51	81				
4. Average GPA	3.2	0.2	18	62	51			
5. Tuition	18450	9686	12	66	63	51		
6. University	0.6	0.5	38	−21	−18	−17	02*	
7. Private	0.5	0.5	−13	48	40	50	82	−14

To examine which factors predict admission, we conducted a series of ordinal logistic regressions, modeling the outcomes of being waitlisted and accepted as ordered improvements over the reference group of rejected applicants. Table 6 summarizes these models, showing how the average GPA and applicants' relative GPA influence admissions outcomes. In each of these models, we hypothesize that applicants' relative GPAs will be positively associated with admission indicating that above average performances are rewarded, but that the average GPA at the applicant's alma mater will also be positive and significantly related to admission. This would represent an error, because, holding all else equal, graduate schools should not want to reward candidates solely for graduating from institutions with more lenient grading norms.

**Table 6:** Summary of ordinal logistic models predicting candidates' admission outcomes (Study 3).

	Model 1	Model 2	Model 3	Model 4	Model 5	Model 6	Model 7
	B (SE)	B (SE)	B (SE)	B (SE)	B (SE)	B (SE)	B (SE)
Deny | Waitlist	0.571***	6.860***	8.500***	10.359***	7.897***	8.305***	9.173***
	(0.016)	(0.045)	(0.022)	(0.037)	(0.026)	(0.050)	(0.024)
Waitlist | Accept	0.840***	7.370***	9.027***	10.913***	8.448***	8.884***	9.544***
	(0.016)	(0.049)	(0.028)	(0.042)	(0.032)	(0.055)	(0.027)
Performance: Relative GPA	0.868***	0.771***	0.733***	0.798***	0.972***	1.057***	1.562***
	(0.042)	(0.068)	(0.073)	(0.075)	(0.078)	(0.081)	(0.058)
Situation: Average GPA	1.389***	0.359*	0.250	0.323	0.736***	0.878***	1.597***
	(0.097)	(0.179)	(0.197)	(0.203)	(0.203)	(0.208)	(0.154)
Interview		1.012***	1.021***	1.077***	1.025***	1.080***	
		(0.030)	(0.030)	(0.031)	(0.031)	(0.032)	
Years of work experience		−0.002	−0.004	−0.075***	0.029**	0.026	0.123***
		(0.009)	(0.009)	(0.012)	(0.011)	(0.015)	(0.012)
GMAT		0.010***	0.013***	0.014***	0.014***	0.015***	0.016***
		(0.000)	(0.000)	(0.000)	(0.000)	(0.000)	(0.000)
School Quality: US News Rating		0.015***	0.011***	0.011***	0.015***	0.014***	0.018***
		(0.002)	(0.002)	(0.002)	(0.002)	(0.002)	(0.002)
Age			0.003	0.035***	0.006	−0.024*	−0.061***
			(0.006)	(0.008)	(0.008)	(0.011)	(0.008)
Gender: Male			−0.614***	−0.648***	−0.696***	−0.714***	−0.560***
			(0.056)	(0.057)	(0.057)	(0.059)	(0.041)
Citizenship: US			0.213*	0.241*	0.248*	0.240*	0.086
			(0.098)	(0.101)	(0.100)	(0.104)	(0.074)
Race: African American			1.266***	1.406***	1.496***	1.643***	1.547***
			(0.144)	(0.151)	(0.148)	(0.154)	(0.107)
Race: Asian			−0.154*	−0.174*	−0.205**	−0.214**	−0.191***
			(0.068)	(0.070)	(0.070)	(0.072)	(0.050)
Race: Hispanic			1.229***	1.280***	1.374***	1.422***	0.943***
			(0.152)	(0.158)	(0.155)	(0.162)	(0.101)
Race: American Indian			0.523***	0.474***	0.640***	0.655***	0.648***
			(0.001)	(0.003)	(0.002)	(0.005)	(0.004)
Race: Other			−0.121	−0.143*	−0.126	−0.122	−0.146**
			(0.070)	(0.073)	(0.072)	(0.075)	(0.055)
Application Years				Included		Included	Included
Admitting School					Included	Included	Included
N	19503	8681	8674	8674	8674	8674	17504
AIC	31774	13323	13032	12598	12627	12208	22235

*p<05; ** p<01; *** p<001.

Models 1 through 3 build an increasingly complex model of admissions decisions starting with only our central variables of interest, and then adding the relevant academic and demographic terms. In all three models, relative GPA is positive and significant, indicating that individual performance is rewarded by increased rates of acceptance. Average GPA is significant and positive as hypothesized in model 1, but this simple model explains a very small amount of the variance in admissions outcomes. In model 3, the Average GPA term is not significant. This is likely because models 1 through 3 do not control for the differences in application year and admitting school. Admission rates vary dramatically across these situations, from 16% to 39% by school, and from 20% to 33% by application year. Subsequent models address this shortcoming.

Models 4 and 5 introduce dummy variables for the four admitting schools and eleven years in which admission decisions were made, while model 6 uses both factors to control for the significant variation in baseline acceptance rates. Model 6 shows that both a candidate's relative GPA and the average GPA at their alma mater have a significant positive effect on their likelihood of acceptance.

Model 7 replicates the findings of model 6 but drops the candidate's interview scores from the equation. After including all of the control variables available in our dataset, the interview score restricts our sample size more than any other control. Dropping it in model 7 allows us to double our sample size and maintain a representative sample through more restrictive analyses. Comparing model 7 to model 6 reveals no qualitative changes in any of the predictors although the relative influence of average GPA increases compared to relative GPA. Further models discussed will generally be adaptations of model 7.

Table 7 presents models 8 through 11, which demonstrate the robustness of our results to three different measures of applicant's undergraduate school quality: US News ratings, Gourman [37] ratings, and the average entrance SAT scores of matriculating students. To compare the relative influence of these three predictors, each was standardized. The relationship between school quality and applicant's admission is critical to the interpretation of our results because of the correlation between quality and average GPA. It is tempting to consider whether the large positive effect of average GPA is related to the fact that many of those students benefiting are from good schools with high grading norms. Models 8–11 show that no matter how school quality is measured, average GPA has a distinct and positive impact on candidate outcomes.

**Table 7:** Summary of ordinal logistic models of graduate school admissions, comparing standardized measures of applicants' undergraduate institution quality (Study 3).

	Model 8	Model 9	Model 10	Model 11
	B (SE)	B (SE)	B (SE)	B (SE)
Deny | Waitlist	7.847***	8.050***	7.829***	7.656***
	(0.023)	(0.022)	(0.023)	(0.024)
Waitlist | Accept	8.218***	8.419***	8.201***	8.026***
	(0.026)	(0.025)	(0.026)	(0.027)
Performance: Relative GPA	1.562***	1.419***	1.529***	1.584***
	(0.059)	(0.055)	(0.057)	(0.060)
Situation: Average GPA	1.597***	2.274***	1.627***	1.556***
	(0.156)	(0.130)	(0.143)	(0.158)
Years of work experience	0.123***	0.132***	0.125***	0.126***
	(0.012)	(0.011)	(0.011)	(0.012)
GMAT	0.016***	0.017***	0.016***	0.016***
	(0.000)	(0.000)	(0.000)	(0.000)
Standardized School Quality: US News	0.294***			0.261***
	(0.026)			(0.038)
Standardized School Quality: Gourman		0.055*		−0.055*
		(0.023)		(0.027)
Standardized School Quality: SAT			0.280***	0.085*
			(0.026)	(0.039)
Age	−0.061***	−0.072***	−0.064***	−0.064***
	(0.008)	(0.008)	(0.008)	(0.009)
Gender: Male	−0.560***	−0.605***	−0.554***	−0.543***
	(0.041)	(0.040)	(0.041)	(0.042)
Citizenship: US	0.086	0.133	0.090	0.044
	(0.074)	(0.071)	(0.072)	(0.074)
Race: African American	1.547***	1.605***	1.577***	1.500***
	(0.107)	(0.103)	(0.105)	(0.111)
Race: Asian	−0.191***	−0.204***	−0.212***	−0.181***
	(0.050)	(0.049)	(0.049)	(0.051)
Race: Hispanic	0.943***	0.965***	0.921***	0.936***
	(0.101)	(0.098)	(0.099)	(0.102)
Race: American Indian	0.648***	0.802***	0.726***	0.565***
	(0.004)	(0.005)	(0.004)	(0.003)
Race: Other	−0.146**	−0.145**	−0.156**	−0.153**
	(0.055)	(0.053)	(0.054)	(0.056)
Application Years	Included	Included	Included	Included
Admitting School	Included	Included	Included	Included
N	17504	18441	18121	16926
AIC	22235	23632	23123	21507

* p<05; ** p<01; *** p<001.

In each of the other models presented, only the US News ratings are included as the measure of school quality for two reasons. First, of the three measures, US News ratings are most closely related to the public reputation of the schools. We expect that a school's reputation influences the evaluation of their alumni more than the less visible educational values captured by the other measures. The data support these inferences. While each of the quality predictors are significant when included alone, model 11 shows that effect of school quality as measured by the US News ratings are more stable in both significance and magnitude.

**Figure 1:**
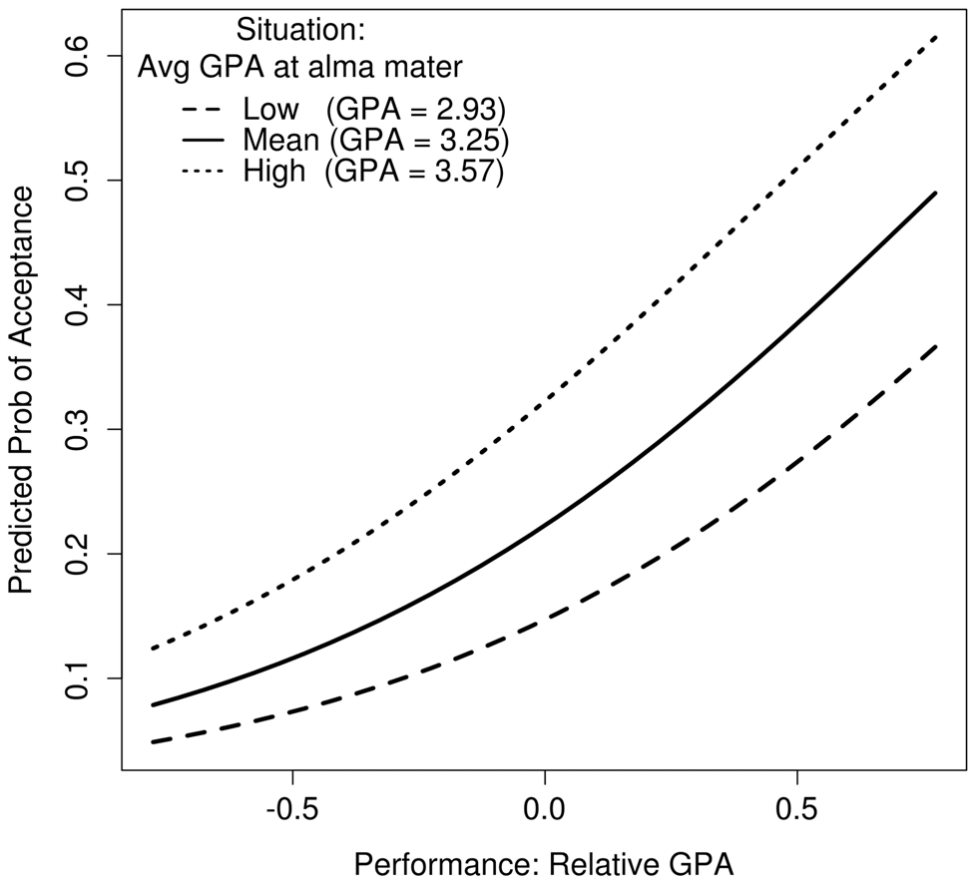
Model 7 predicted probability of acceptance by situation and performance (Study 3).

Table 8 presents three new models to address alternative analysis approaches and concerns. Model 7 is presented again for ease of comparison. Model 12 includes an interaction between the two primary predictors, relative and average GPA. The interaction is significant and positive, but it does not affect the results from the main effect which remain positive, significant, and of similar magnitude. The positive coefficient on the interaction term means that the effect of average grades on the probability of admission is larger for above-average candidates than those performing below their local average as illustrated in Figure 1.

**Table 8:** Summary of alternative analysis approaches (Study 3).

	Model 7	Model 12	Model 13	Model 14
	B (SE)	B (SE)	B (SE)	B (SE)
Deny | Waitlist	9.173***	9.155***	8.333***	14.244***
	(0.024)	(0.024)	(0.040)	(0.016)
Waitlist | Accept	9.544***	9.527***	8.691***	14.616***
	(0.027)	(0.027)	(0.043)	(0.020)
Performance: Relative GPA	1.562***	1.568***	1.685***	
	(0.058)	(0.058)	(0.075)	
Performance: Nominal GPA				1.562***
				(0.051)
Situation: Average GPA	1.597***	1.397***	1.715***	0.035
	(0.154)	(0.148)	(0.217)	(0.146)
Relative GPA x Average GPA		1.250***		
		(0.032)		
Years of work experience	0.123***	0.125***	0.130***	0.123***
	(0.012)	(0.012)	(0.015)	(0.012)
GMAT	0.016***	0.016***	0.015***	0.016***
	(0.000)	(0.000)	(0.000)	(0.000)
School Quality: US News Rating	0.018***	0.018***	0.017***	0.018***
	(0.002)	(0.002)	(0.003)	(0.002)
Age	−0.061***	−0.062***	−0.081***	−0.061***
	(0.008)	(0.008)	(0.011)	(0.009)
Gender: Male	−0.560***	−0.556***	−0.511***	−0.560***
	(0.041)	(0.041)	(0.049)	(0.042)
Citizenship: US	0.086	0.078	−0.154	0.086
	(0.074)	(0.074)	(0.092)	(0.074)
Race: African American	1.547***	1.567***	1.439***	1.547***
	(0.107)	(0.107)	(0.134)	(0.106)
Race: Asian	−0.191***	−0.190***	−0.181**	−0.191***
	(0.050)	(0.050)	(0.058)	(0.050)
Race: Hispanic	0.943***	0.953***	0.986***	0.943***
	(0.101)	(0.101)	(0.124)	(0.101)
Race: American Indian	0.648***	0.669***	0.697***	0.648***
	(0.004)	(0.004)	(0.004)	(0.003)
Race: Other	−0.146**	−0.143**	−0.078	−0.146**
	(0.055)	(0.055)	(0.066)	(0.055)
Application Years	Included	Included	Included	Included
Admitting School	Included	Included	Included	Included
N	17504	17504	11361	17504
AIC	22235	22219	15018	22235

** p<01; *** p<001.

Model 13 tests the analysis with a subset of the sample of applicants. Although we maintain that it is normatively ideal for admissions decision makers to discount applicants' nominal GPAs to account for variation in grading norms, this may be difficult to do in practice. It was quite difficult for us to obtain average GPA data from the wide selection of undergraduate institutions represented in our sample of applicants. Our sample of professional admissions workers from Study 1 indicated that they had some information about grading norms for nearly two thirds of applicants. Because it is likely that admissions staffers are more familiar with the top undergraduate institutions, we restricted the sample in model 13 to those applicants who graduated from schools in the top quartile of US News ratings. This decreased the sample by only 35% compared to the model 7 because so many applicants in the sample graduated from these top institutions. The model shows that the applicant's GPA continues to be significant and positive, but that even in this top quartile, average GPA is robustly significant and positive. In fact, in this subset, the magnitude of the effect of average GPA is slightly larger than that of the candidate's relative performance. All else equal, applicants who are a tenth of a point above average at their school are no more likely to be accepted than applicants that had average GPAs at a school where the grading norms were a tenth of a point higher than other schools.

Finally, in model 15 we ensure that our conclusions are robust to an analysis without our computed relative GPA measure. In this model, normative admissions decisions would discount situational influences by weighting average GPA negatively and to a similar degree that nominal GPA is positive. We see that average GPA is not significantly different than zero, consistent with the conclusion that decision makers are not adjusting nominal performance in light of information about the situation.

#### Discussion

We argue that the tendency for admissions decisions to ignore grading leniency is explainable by attributional processes that take performance at face value and fail to discount it by situational influences. The result is that applicants from schools with lenient grading are evaluated more positively simply because their GPAs are higher. Model 8 shows that applicants benefitting from an average GPA one standard deviation above the mean (just 0.17 points) are 31% more likely to move up from denied to waitlisted or from waitlisted to accepted. Figure 1 illustrates the magnitude of this effect.

However, the field data do not allow us to rule out two viable alternative explanations for our results. First, it might not be fair to assume that admissions staffs have perfect information about average grades at all other academic institutions. After all, these data were costly for us to collect. Without the data, it would be difficult for admissions departments to use the information to discount grades appropriately, and they would simply have to rely on absolute GPAs as useful (if imperfect) measures of academic performance. On the other hand, this information is critical to their ability to interpret applicants' GPAs, and admissions offices are likely to have better ways of obtaining these data than did we. A failure to demand it, in and of itself, suggests a failure to appreciate its value.

Second, it is possible that average grades capture something important about the undergraduate institution. Although we considered the roles of institution quality, public/private status, tuition, and student quality as control variables, it is possible that lenient grading may covary with other important features of institutions that are not captured in our control variables. The experimental data from the first study can help us to address this concern to some degree. Professional admissions workers evaluated candidates from various fictional schools whose grading leniency varied substantially but that were equal in quality and selectivity. This makes it unlikely that our participants favored candidates from lenient-grading institutions because they believed that the students were actually better at lenient-grading institutions.

## General Discussion

Many studies in the social psychology and organizational behavior literatures have found that people tend to attribute too much influence to disposition and too little influence to situational factors impinging on the actor when explaining others' behaviors, achievements, and failures. This common tendency, labeled the correspondence bias or the fundamental attribution error, has been shown to be robust across a variety of contexts and situations. Yet, to date, most of the evidence about this bias comes from laboratory experiments with college students as participants, and its implications for field settings and organizational outcomes are seldom examined. Using data from both the experimental laboratory and the field, we extend prior research by investigating whether this tendency leads experienced professionals to make systematic mistakes in their selection decisions, favoring alumni from academic institutions with higher grade distributions and employees working in favorable business climates. Our results indicate that candidates who have demonstrated high performance thanks to favorable situations are more likely to be rated highly and selected. Across all our studies, the results suggest that experts take high performance as evidence of high ability and do not sufficiently discount it by the ease with which that performance was achieved. High grades are easier to achieve in an environment where the average is high and so are less indicative of high performance than are the same grades that were earned from an institution with lower grades on average. Sky-high on-time percentages should be less impressive at an airport that was running well before the manager got there. Although we focused on two selection scenarios, we believe the results speak to other selection and evaluation problems.

Indeed, we see consistent evidence of situation neglect in contexts where political and business leaders are credited with performance that derives directly from stochastic economic factors. Voters face a Lewinian dilemma when they evaluate the performance of incumbent politicians running for re-election. They should reward politicians who create positive change for their constituencies while considering what portion of those changes were due to lucky or exogenous factors. Wolfers [41] finds that voters, like our admissions professionals and executives, favor politicians that had the good luck to work under favorable conditions. Voters are more likely to reelect incumbents after terms marked by positive national economic trends or (in the case of oil-rich states) high oil prices. CEOs also benefit from fortuitous economic conditions for which they are not responsible. Bertrand and Mullainathan [42] present evidence that CEO compensation is driven to equal degrees by their management and the uncontrollable economic conditions in which they managed. Stakeholders in these cases have strong incentives to reward leaders who add value above the vagaries of the economy, but they seem blind to the difference.

It is often the case that structural and situational factors are the most powerful influences on behavior. Within organizations, for example, it is easier to succeed in some jobs than in others [43]. Sometimes people will achieve positive outcomes simply because of a beneficent environment. It is easier to achieve success as a manager when your team is strong than when your team is weak. Likewise, it is easier to obtain a strong education in an excellent private school than in an under-funded public school. And it is easier to achieve high grades at schools where higher grades are the norm. So it would be a mistake to neglect situational effects on performance, but that is what our data suggest that even experts and professionals tend to do.

Are we always doomed to make erroneous correspondent inferences? Evidence suggests not; the bias is subject to a number of moderating factors. These are useful to consider both because they provide clues about the psychological mechanisms at work and because they suggest potential debiasing treatments. For instance, when people are stressed, distracted, or busy, they are more likely to fall victim to the correspondence bias [44]. Those with greater capacity for reflective thought, as measured by need for cognition, are less likely to show the bias [45]. When people feel accountable to others, they are less likely to show the bias [46]. When people are in good moods, they appear more likely show the bias [47]. And some collectivistic cultures may be less vulnerable to the correspondence bias than individualistic ones [48], [49].

Organizations often adopt practices because they are legitimate, popular, or easy to justify [50], [51]. That may help explain why we observed such consistency in admissions policies in neglecting to consider differences in grade distributions between institutions. This sort of consistency in organizational “best” practices can create incentives for individuals to play along, despite their imperfections. Indeed, it is even conceivable that cultural or linguistic norms can make it easier for individuals to follow decision norms that are more easily understood by or explained to others. On the other hand, it is reasonable to assume that finding a better system to evaluate applicants would improve admissions decisions, allowing the schools that do it to identify strong candidates that other schools neglect. The Oakland Athletics baseball team did just this when it pioneered a new statistical approach to identifying promising baseball players to recruit [52]. Their success has since been emulated by other teams, changing the way baseball's talent scouts pick players. However, the problem for admissions departments may be more complicated because explicitly tarring some institutions as lenient-grading is likely to elicit energetic protests if they ever find out about it [53].

It is common in organizations for the abilities of an individual, a department, or a division to be shrouded in complicating or confounding influences that make them difficult to detect or measure [54]. Indeed, as much as ratings systems like grades and performance metrics like on-time percentages can help clarify standards for evaluation, they can also be used to obscure performance [55]. Variation in grading standards between institutions obscures the value of using grades to measure student performance. It is probably in the interest of lenient-grading institutions to hide the degree of their leniency. Consistent with this motive, recent years have seen changes in the disclosure that institutions are willing to make [56]. Fewer academic institutions are willing to disclose average grading data or class rankings for their students or alumni. When we contacted institutions to inquire regarding average grades elite, expensive, private institutions – those with the highest average grades – were most likely to decline to disclose the information.

### Organizational Image, Legitimacy, and Stakeholder Appraisals

The strategic use of scoring and assessment metrics has implications at the organization level because of the way that institutions compete. Scott and Lane [57] advanced a theory of organizational image in which stakeholders (both members as well as outside audiences) play a key role in shaping the organization's image by making legitimacy appraisals that can counterbalance the organization's attempts at image management. This model is built on the dual premises that organizations and their members derive personal and economic benefits from promoting a positive image [58], [59], but that salient audiences have a role in validating that image [60], [61]. These forces form an equilibrium that balances the organization's incentives for an unbounded positive spin with the utility gained by stakeholders from an image grounded in reality. Scott and Lane [57] term the specific mechanism by which this equilibrium is reached *reflected stakeholder appraisals*. In the present paper we have investigated a setting in which stakeholders may have difficulty judging the appropriateness of image-relevant information which could then threaten the stability of the reflected stakeholder appraisal equilibrium.

In the context of higher education, graduating students are among the primary interfaces through which employers, graduate schools, and communities interact with undergraduate institutions. Their reputation in the form of grades contributes to the reputation [62] of the organization. As such, undergraduate institutions have an incentive to promote an image of intelligence and achievement to these outside audiences by maintaining a relatively high grade distribution. Given the tremendous value of being able to place alumni in better graduate schools and in better jobs, universities cannot be expected to go too far in seeking to curtail grade inflation. For example, universities are unlikely to implement meaningful institutional changes such as replacing grades with percentile rankings. Instead, we should expect academic institutions to pay lip service to the importance of high academic standards while at the same time avoiding publicizing average grade distributions and avoiding reporting class rank data on their students.

Do we see unchecked escalation of grade distributions by a market full of organizations unconstrained by the critical feedback from shareholders? Of course, there are multiple mechanisms supporting a moderate equilibrium even without functioning shareholder criticism of the type we have described, but some data suggest grade inflation is a prolonged and significant trend in U.S. Education [6]. More troubling are anecdotal reports of institutions manipulating their grade distribution with the publicly expressed intent of influencing the selection decisions of hiring firms [63]. Clearly, these institutions are anticipating that employers will not sufficiently discount the grades of their alumni to eliminate the advantage their inflated grades will confer.

### Limitations and Directions for Future Research

Our studies are subject to several important limitations. First, the sample used in our first study was relatively small due to the size of the admissions department that participated, even though the results were highly significant. In addition, the first and second studies employed hypothetical decisions, which may have limited validity as a model of fully consequential and incentivized decision making. Future research could benefit from a more qualitative research approach to investigate how admissions and promotion decisions are made by various organizations. As for Study 3, there are many variables (such as variations in average GPA by discipline within a school) for which we did lacked information and thus could not control in our analyses. These variables may have important influences on admission decisions that are not captured in the present research. Although these are important limitations, it is also worth noting that the limitations differ across studies and yet the findings are robust.

The conclusions implied by our results as well as the limitations of our research bring forth some fruitful and interesting possible avenues for future research. One interesting question is whether other academic selection contexts would show the same patterns as business school admissions decisions. Law schools, for instance, use the Law School Admissions Council, an organization that (among other things) processes applications for law schools and provides a service that gives schools a sense of where a given applicant's GPA falls relative to other applicants that the LSAC has seen from that same institution. The Graduate Management Admissions Council does not process business school applications and so does not provide an equivalent service for business schools. Does the LSAC's assistance help law schools make better admissions decisions?

Similarly, future research could explore the implications of the correspondence bias for promotions of business professionals. Just as educational institutions vary with respect to the ease of achieving high grades, so do companies, industries, and time periods differ with respect to the ease of achieving profitability. There are some industries (such as airlines) that are perennially plagued by losses and whose firms have trouble maintaining profitability. There are other industries (such as pharmaceuticals) that have seen more stable profitability over time. And clearly there are changes over time in industry conditions that drive profitability; for example, global oil prices drive profitability among oil companies.

We believe an important avenue for further investigation lies in continuing the study of the correspondence bias in empirical settings with organizationally-relevant outcomes. A more thorough understanding of the implications of this common bias for organizations could be achieved by further investigating business decisions such as promotions. There are also a multitude of other business decisions in which a latent variable of interest is seen in the context of varying situational pressures. Investment returns, sports achievements, and political success are all domains in which judgments are vulnerable to the tendency to insufficiently discount the influence of the situation. We expect that the correspondence bias affects outcomes in these domains.

Our theory holds that a firm's good fortune (in the form of greater profits) will be mistaken as evidence for the abilities of its managers. If this is so, then we should more often see employees of lucky firms being promoted than of unlucky firms [64]. We would expect, for instance, that pharmaceutical executives are more likely to be hired away to head other firms than are airline executives. However, this finding might be vulnerable to the critique that pharmaceutical executives actually are more capable than are airline executives–after all, their firms are more consistently profitable. Therefore, a better way to test this prediction would be using an industry (such as oil) in which fortunes fluctuate over time due to circumstances outside the control of any firm's managers. Our prediction, then, would be that oil executives are more likely to be hired away to head other firms when the oil industry is lucky (i.e., oil prices are high) than when the industry is unlucky (i.e., oil prices are low).

### Theoretical Contributions

Our results contribute to the literature on the psychological process at work in comparative judgment, a literature that stretches across psychology [65], economics [66], and organizational behavior [67]. In this paper, we extend previous research by examining judgmental contexts in which expert decision-makers are comparing outcomes that vary with respect to both nominal performances and their ease. We should also point out that these results are, in a number of ways, more dramatic than the results of previous research showing biases in comparative judgment. Previous results have been strongest when participants themselves are the focus of judgment [65], [68]. Biases in comparative judgment shrink when people are comparing others, and shrink still further when they have excellent information about performance by those they are comparing [69]. Biases disappear when comparisons are made on a forced ranking scale [70]. In this paper, we have shown comparative judgments to be powerfully biased even when people are evaluating others about whom they have complete information (as modeled in Study 1), and even when the assessments (e.g., admission decisions) are made on a forced distribution that prevent them from rating everyone as better than everyone else.

Although attribution biases have been extensively studied, the vast majority of this research has been conducted with convenience samples of students. This raises the familiar concern that deviations from normative decision outcomes are the result of insufficient motivation, or that they could be corrected with sufficient experience. Indeed, some studies have found that experts often employ superior decision strategies and enjoy more positive outcomes [71]. Our results however, suggest that experts do not discount nominal performance in light of information about the situation any differently than students in a hypothetical admissions task [35]. The robustness of this effect in expert populations connects a well-known psychological phenomenon to a world of selection decisions with tangible and troubling implications.

Finally, our work contributes to research on human resource and employment decisions. Previous research has examined a number of factors that influence employment decisions such as hiring and promotion. For instance, research suggests that objective applicant qualifications, such as education or work experience, play a large role in hiring and selection decisions [72], [73] but are often subject to situational factors. One of the qualifications previous studies have examined is applicants' general mental ability, which is often measured using the applicants' GPA. Consistently, research has demonstrated that an applicant's GPA influences evaluation and subsequent hiring decisions [74], [75]. Our results contribute to this literature by suggesting that future studies may benefit from also including information about the average GPA of the institution where the applicant studied, or other appropriate measures of situational influence.

### Conclusion

Each of our studies supports the hypothesis that people rely heavily on nominal performance (such as GPA) as an indicator of success while failing to sufficiently take into account information about the distributions of performances from which it came. To the extent that admissions officers and hiring managers generally show the same biases we found, graduate programs and businesses are collectively choosing to select candidates who demonstrated their merit in favorable situations rather than selecting the best candidates. The consequences could be substantial for both the sufficiently qualified but unselected candidates as well as for the organizations that systematically select lower performing candidates than they could.
